# Open-Joint Navicular Sheath1Read before the Niagara and Orleans County Veterinary Medical Society, August 16, 1901.

**Published:** 1901-09

**Authors:** J. O. Moore

**Affiliations:** Wilson, N. Y.


### OPEN-JOINT NAVICULAR SHEATH.[1]

By J. O. Moore, V.S.,
WILSON, N. Y.

On June 23, 1901, I was called to see a lame mare. On arrival I was informed that two weeks previous the owner had extracted a large wire nail, and the horse had remained lame ever since, and he could not see why it did not get better after he had removed the nail.

When I looked at the patient I found her lying down on the left side and in very much pain.

On examination I found a heavy discharge of a straw-colored fluid and that the nail had entered the cleft of the frog about the centre, and on calling for the nail I could see that it had penetrated very deeply. I immediately stripped off the insensitive frog and found the sensitive frog swollen and bulging out all around the nail hole, and on using a little pressure on the heel there was a synovial discharge from the opening. I then diagnosed the case as open joint of the os navicular joint.

My treatment the first day was cleansing the foot and frog with peroxide of hydrogen, then washing with carbolized water. I used a syringe in the opening as much as I could, but it was so sensitive that this was difficult. I then bound up the foot in a bran poultice, and ordered it kept moist with water until I returned the next morning.

On my second visit I found her still suffering much pain ; her pulse was 60 and good and strong. Temperature 100°, as the day before. On removing the poultice I found there had been quite a discharge of synovia and pus on the bran, but the frog had cleaned up. I washed and dressed the foot as the day before, and then proceeded to put the mare in a sling, but this she objected to, and as soon as they were adjusted she would lie down. After trying to prevent her from doing this for about an hour, I took

[1] Read before the Niagara and Orleans County Veterinary Medical Society, August 16, 1901.

them off, and had them spade up a large plot of ground under a tree, and removed her there and enclosed her with a slat fence. I continued the treatment for a week. I then saw her, and could see no improvement in lameness, and also found a large swelling in the heel, with very little discharge from the frog. I then lanced the heel, let the pus escape, and syringed it out with peroxide of hydrogen, cleaned and dressed the frog with compound tincture of benzoin, and had this treatment continued for another week, with poulticing as before.

On my third visit I found no discharge from the frog, the heel closed up, and the mare able to put her toe on the ground. The insensitive frog was beginning to form. I discontinued the poultice, and applied a blister around the coronary band and had her stood (after the blister was washed off) in mud up to the hair on the hoof. This I continued for ten days, then applied a spring shoe with a straight bar across the heels and a spring from the toe to the bar, so that no part of the sole or frog touched the ground. I also applied another blister, and saw her yesterday, and found her travelling around with lameness scarcely perceptible.

